# Pediatric Acute Liver Failure

**DOI:** 10.3390/pediatric15030039

**Published:** 2023-07-14

**Authors:** Claudia Mandato, Pietro Vajro

**Affiliations:** Department of Medicine, Surgery and Dentistry “Scuola Medica Salernitana” University of Salerno, 84081 Baronissi, Salerno, Italy

Pediatric acute liver failure (PALF) has recently become a subject of great interest when multiple clusters of non-A to non-E severe acute hepatitis in otherwise healthy young children with a median age of 2 years were reported around the world. Approximately 10% of these cases required liver transplantation (LT) and several died due to ALF. As the etiopathogenic and/or contributing factors were controversial, this condition has been named Childhood Severe Acute Hepatitis of Unknown Origin (AHUO) [1,2]. Data from three studies published in Nature (two from the UK and one from the USA) [3,4,5] have now provided quite convincing new proofs of adenoviral involvement. According to them, high levels of replication products from adeno-associated virus 2 (AAV2), aided by an adenovirus (HAdV) and, in severe cases, also by human herpesvirus 6B (HHV-6B), may have been responsible for triggering an immune-mediated hepatic disease in genetically and immunologically predisposed children [3,4,5,6,7]. These events could have been eased due to the reduced spread of common viral infections as a consequence of physical distancing during the COVID-19 pandemic [1,8]. The exact pathogenetic mechanisms, however, still remain unclear [9]; no one knows when and where future outbreaks of this severe hepatitis will occur, and if they will cause other PALF cases.

What is ALF and why is it still so alarming? ALF is a rapidly progressive condition with a high mortality rate without LT. It is characterized by the sudden onset of liver test abnormalities representative of hepatocellular damage and coagulopathy. In children, the efforts to characterize this condition are recent: the most agreed definition of pediatric ALF (PALF) was established in 1999 by a North American and British pediatric ALF study group (PALFSG) [10]. Characteristically, PALF should present with no known evidence of pre-existing chronic liver disease, and—different from adults—hepatic encephalopathy (HE) is not required to make the diagnosis (Table 1).

The results from a prospective, multi-center case study collecting demographic, clinical, laboratory, and short-term outcome data of children from birth to 18 years with the above definition confirmed that, although clinical encephalopathy may not be present in children, the highest mortality was observed in patients with severe HE or demonstrating HE progression, and outcomes varied between patient sub-groups [11,12]. Although the causes of PALF may be subdivided into infectious, immunologic, metabolic, ischemic, and toxin- or drug-related, a significant proportion of cases (more than one in three) still have an unknown etiology (indeterminate PALF), reaching a proportion of two in three among subjects aged 1–5 years [13] despite advances in diagnostics [14,15]. According to a recent systematic review [16], in developing countries, hepatitis A represents the leading cause of PALF at all ages. In endemic areas, Dengue and yellow fever should also be considered. In developed countries (Figure 1), an age-based diagnostic approach can help to identify the possible underlying cause, administer the appropriate life-saving therapy, and assess the indications for LT. Drug toxicity, toxin ingestion, accidental or intentional drug overdose [e.g., acetaminophen (the most common drug involved), tetracycline, ecstasy, toxic mushroom Amanita phalloides, herbal and dietary supplements, etc.] are also frequent causes, particularly in adolescents.

Due to the rapid deterioration of LFTs, diagnostic investigations need to be combined with equally rapid measures to detect and treat a vast array of common complications of PALF (Table 2).

Recently, in close succession, the Italian [17], American [18], and European [19,20] societies for pediatric gastroenterology, hepatology, and nutrition have issued new recommendations regarding the etiology, clinical features, diagnostic work-up, medical management, and transplant indications for newborns, infants, and children with ALF. All recommendations used the 1999 PALFSG’s definition of PALF [10,11,12]. Because severe acute liver injury can cause significant disease and results in death or the need for LT, NASPGHAN [18] has emphasized that optimal treatment should be based on early transfer to or contacting an experienced pediatric liver transplant center, with age-appropriate intensive clinical care, while monitoring and managing common renal, neurological, metabolic, pulmonary, and nutritional complications regardless of the etiology. Efforts should be made to obtain support from an interactive team of hepatologists, critical care specialists, and liver transplant surgeons to make correct LT-timing decisions when necessary.

ESPGHAN [19,20] underscores that the knowledge gained from adult patients in the intensive care unit cannot necessarily be transferred to children. In fact, the diagnosis is strongly age-dependent, with newborns and infants being at higher risk of poor outcomes unless they receive prompt medical attention, proper imaging, appropriate critical care, and timely and judicious referral to an LT center. Appropriate cardiovascular support, brain imaging, and acquiring electroencephalograms are essential first steps in management of PALF. The guidelines suggest that early detection of encephalopathy, and use of ammonia-lowering drugs can improve the prognosis. The associated acute renal failure, fluid overload, and severe electrolytes imbalance require continuous renal replacement therapy which is preferred to intermittent hemodialysis. Moreover, unnecessary correction of coagulopathy in patients without bleeding can be detrimental. Specifically, in regard to LT, it appears that current scoring systems may not reliably predict mortality and the timing of LT in a child with ALF should be individualized. Auxiliary LT and human hepatocyte transplantation could help restore the native liver in certain circumstances in expert centers. The health-related quality of life needs attention as it remains impaired in children after ALF in the first year after hospitalization [20]. Both NASPGHAN and ESPGHAN societies highlight the importance of an etiology-based therapies for some conditions with specific treatments when available (Table 3).

Several gray areas still remain for conditions such as (a) Acute on Chronic Liver Failure, e.g., in cases with an acute hepatic insult superimposed on autoimmune hepatitis or Wilson disease, or viral reactivation in chronically HBV-infected children, and (b) congenital disorders of intracellular trafficking (e.g., NBAS, SCYL1, or RINT1) in which the patients may develop recurrent PALF that has no specific treatment. Mitochondrial disorders also remain particularly worrisome as, due to occult multisystem involvement, progressive extra-hepatic disease can occur after LT.

In conclusion, after the initial characterization of the patient presentation, proper patient management needs to be conducted along multiple parallel paths. Evaluation of the cause of PALF, a process guided by the patient’s age giving priority to the diagnosis of treatable disorders, should not delay monitoring of the function of each organ system to identify and treat complications and provide medical support to maximize health and survival. All these interventions may result in increased transplant-free survival and improved post-LT outcomes, thereby reducing mortality and morbidity [21]. Future studies should aim to better characterize the mechanisms of PALF that could provide therapeutic opportunities, validate prognostic modeling scoring systems [21], and help to develop new mechanistic and clinical models to examine treatment strategies and transplant decisions which will further improve outcomes. These include effective bridges to recovery that are presently available (e.g., hepatocyte transplantation) [22,23]. Last, but not least, what about Childhood Severe AHOU ? Is this a real and enduring threat for our children? Much probably time will tell. [24] In any case, should cases recur, early diagnosis along with adenovirus testing [2] and timely referral to centers with available liver transplant facilities [8] remain crucial due to the potential for death from ALF. 

## Figures and Tables

**Figure 1:**
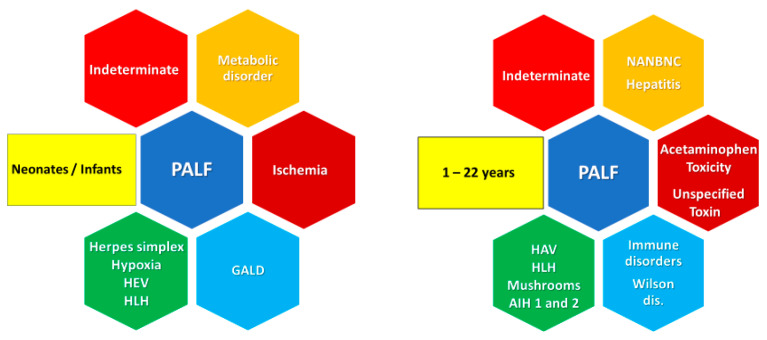
Main causes of pediatric acute liver failure in neonates/infants and older children. AIH, autoimmune hepatitis; GALD, gestational alloimmune liver disease; HEV, hepatitis E virus; HLH, hemophagocytic lymphohistiocytosis; NANBNC, non-A–non-B–non-C hepatitis virus; PALF, pediatric acute liver failure.

**Table 1:** PALFSG diagnostic criteria for pediatric acute liver failure with/without evidence of hepatic encephalopathy (all three components required) [10].

1. Acute onset liver disease with no evidence of pre-existing liver disease within the preceding 8 weeks of presentation.
2. Biochemical evidence of severe liver injury.
3. Coagulopathy not corrected by vitamin K: a. Prothrombin time (PT) ≥ 15 s or INR ≥ 1.5 with evidence of hepatic encephalopathy or b. PT ≥ 20 s or INR > 2.0 with or without encephalopathy.
INR, international normalized ratio; PALFSG, Pediatric Acute Liver Failure Study Group

**Table 2:** Common complications of PALF.

System	Complication
Neurologic	Hepatic encephalopathy; Cerebral injury (cerebral edema);
Hematologic	Coagulopathy; Aplastic anemia
Gastrointestinal	Ascites; Gastrointestinal bleeding; Pancreatitis
Kidney injury	Drugs or toxins; Hypovolemia; Shock; Hepatorenal syndrome
Metabolic	Low levels of glucose, potassium, phosphates; acid-base disturbances.
Infectious	Bacterial infection and sepsis
Cardiopulmonary	Pulmonary edema

**Table 3:** Etiology-based therapies for PALF.

PALF Etiology	Available Treatment
Acetaminophen	N-Acetylcysteine
Tyrosinemia	Nitisinone and protein-restricted diet
Galactosemia, fructosemia	Galactose- and fructose-restricted diets, respectively
Urea cycle disorders	Ammonia scavengers (benzoate and phenylacetate/phenylbutyrate) + protein-restricted diet. Recurrent episodes of PALF are possible
Fatty acid oxidation (FAO) disorders	i.v. glucose infusion; L-carnitine administration; avoidance of i.v. lipid and drugs that inhibit FAO (e.g., valproic acid, NSAIDs, salicylates, etc.)
Viral hepatitis	HAV: no currently available safe and efficient antiviral cure. HEV: has worse outcomes in pregnant women; ribavirin may be useful HBV: antivirals in acute HBV are debatable but are recommended in severe cases /PALF to reduce recurrent HBV risk after LT HSV: has high mortality in the neonatal period; LT in conjunction with antiviral therapy (acyclovir) can be life-saving Other minor hepatotropic viruses: may cause PALF in immunocompromised patients outside the neonatal period. As observed in childhood severe AHUO, they can occur also in immunocompetent children. Treatment: antiviral drugs + LT
Neonatal hemochromatosis/gestational alloimmune liver disease (GALD)	It is an alloimmune process due to maternal alloantibodies that activate the fetal complement cascade attacking the fetal liver. Exchange transfusion and i.v. immunoglobulin (IVIG) allow for LT-free survival in most cases
Hemophagocytic lymphohistiocytosis (HLH)	Primary HLH: hematopoietic stem cell transplantation Secondary HLH: dexamethasone, ciclosporin, and VP-16; rituximab in EBV [+] cases. Targeted T-cell lysis with ATG or alemtuzumab is promising
ATG, antithymoglobulin; i.v., intravenous; NSAID, non-steroidal anti-inflammatory drug; LT, liver transplantation; PALF, pediatric acute liver failure.	
